# Traditional Chinese Medicine Diagnosis “*Yang-Xu Zheng*”: Significant Prognostic Predictor for Patients with Severe Sepsis and Septic Shock

**DOI:** 10.1155/2013/759748

**Published:** 2013-10-24

**Authors:** Sunny Jui-Shan Lin, Yung-Yen Cheng, Chih-Hung Chang, Cheng-Hung Lee, Yi-Chia Huang, Yi-Chang Su

**Affiliations:** ^1^Department of Chinese Medicine, National Defense Medical Center, Tri-Service General Hospital, Taipei 11490, Taiwan; ^2^Graduate Institute of Chinese Medicine, College of Chinese Medicine, China Medical University, Taichung 40402, Taiwan; ^3^School of Chinese Medicine, College of Chinese Medicine, China Medical University, Taichung 40402, Taiwan; ^4^Department of Internal Medicine, Nantou Hospital, Department of Health, Executive Yuan, Nantou 54062, Taiwan; ^5^Rehabilitation Institute of Chicago, Chicago, IL 60611, USA; ^6^Department of Physical Medicine and Rehabilitation, Northwestern University Feinberg School of Medicine, Chicago, IL 60611, USA; ^7^Graduate Institute of Biostatistics, China Medical University, Taichung 40402, Taiwan; ^8^Department of Traditional Chinese Medicine, Taichung Veterans General Hospital, Taichung 40705, Taiwan

## Abstract

Pathogenesis of sepsis includes complex interaction between pathogen activities and host response, manifesting highly variable signs and symptoms, possibly delaying diagnosis and timely life-saving interventions. This study applies traditional Chinese medicine (TCM) *Zheng* diagnosis in patients with severe sepsis and septic shock to evaluate its adaptability and use as an early predictor of sepsis mortality. Three-year prospective observational study enrolled 126 septic patients. TCM *Zheng* diagnosis, Acute Physiology and Chronic Health Evaluation (APACHE) II score, and blood samples for host response cytokines measurement (tumor necrosis factor-**α**, Interleukin-6, Interleukin-8, Interleukin-10, Interleukin-18) were collected within 24 hours after admission to Intensive Care Unit. Main outcome was 28-day mortality; multivariate logistic regression analysis served to determine predictive variables of the sepsis mortality. APACHE II score, frequency of *Nutrient*-phase heat, and *Qi-Xu* and *Yang-Xu Zhengs* were significantly higher in nonsurvivors. The multivariate logistic regression analysis identified *Yang-Xu Zheng* as the outcome predictor. APACHE II score and levels of five host response cytokines between patients with and without *Yang-Xu Zheng* revealed significant differences. Furthermore, cool extremities and weak pulse, both diagnostic signs of *Yang-Xu Zheng*, were also proven independent predictors of sepsis mortality. TCM diagnosis “*Yang-Xu Zheng*” may provide a new mortality predictor for septic patients.

## 1. Introduction

Mortality of severe sepsis and septic shock remains elevated despite progress in therapy [1]. Diagnostic methods reliably identifying patients with a higher risk of death are urgently needed in order to provide timely treatment and improve cost-efficacy of intensive care [2]. Since reliable concepts and accurate measurements to rate mortality risk and stratify severity of septic patients are insufficient [3, 4], a classification system named PIRO was developed to stratify patients on the basis of their *predisposition*, the nature and extent of *insult/infection*, nature and magnitude of *response*, and degree of concomitant *organ dysfunction* [5, 6]. Multiple host and pathogen-associated characteristics are utilized in this system to predict outcome. Similar diagnostic concepts also exist in traditional Chinese medicine (TCM) *Zheng* diagnosis.

The integrity of the human body and its close interaction with the environment (e.g., infectious pathogens) are emphasized in TCM. Disease is considered as a common product of both pathogenic factors and maladjustment in the body [7]. While diagnosing patients, *Zheng* is an outcome after all signs and symptoms are analyzed. As disease progresses, *Zheng* may evolve, since signs and symptoms may change [8]. The TCM *Zheng *diagnosis implies both the subtype categorization and severity staging of disease progress, making TCM *Zheng* diagnosis feasible for adoption as a disease stratification tool in clinical practice [9].

Severe acute respiratory syndrome (SARS) is an infectious disease caused by a novel coronavirus. It is believed that complicated pathogenesis and severity of SARS arise from complex host responses against infectious agents [10]. During the SARS outbreak in China, 40–60% of infected patients received standard modern medical treatment integrated with Chinese medicine treatment [11]. While facing the challenge to treat SARS patients, TCM *Zheng* differentiation enables physicians to prescribe medicine in accordance with the process and nature of the illness [12]. The positive effects of this integrative treatment were reported by WHO and other review articles [11, 13–16].

Our study applied TCM *Zheng* diagnosis in patients with severe sepsis and septic shock to see whether such diagnosis can be adopted as an early predictor of mortality. We also wanted to probe for significant differences between septic patients with and without this predictive TCM *Zheng* with regard to APACHE II score, which measures the deteriorated general condition of patients [17], and some host response cytokines have been reported as closely related to sepsis mortality: tumor necrosis factor-*α*  (TNF-*α*), Interleukin-6 (IL-6), Interleukin-8 (IL-8), Interleukin-10 (IL-10), and Interleukin-18 (IL-18) [18–24]. We also explored each diagnostic sign of predictive TCM *Zheng* for possible adoption individually or in combination with other signs to predict mortality. These predictive signs can then be applied by Western physicians to timely recognize the septic patients at higher risk of death.

## 2. Materials and Methods

### 2.1. Development of TCM Zheng Diagnosis for Severe Sepsis and Septic Shock

First, literature review on TCM *Zhengs* for diagnosing infectious diseases in Chinese classical medicine was completed by the research team. Then four rounds of meetings were held to develop TCM *Zhengs* and diagnostic criteria to classify patients with severe sepsis and septic shock into stages of disease progress. Ten participating experts had both Western and Chinese professional training, medical licenses, more than ten years of clinical experience, and had worked in medical centers.

After several rounds of discussions, two major theories of infectious disease in TCM, *Treatise on Cold Damage Diseases *(*Shanghanlun, 傷寒論*) and *Treatise on Warm Heat Disease* (*Wenrelun, 溫熱論*) became central topics. Experts integrated both these theories to outline pathogenesis of sepsis in TCM and develop TCM *Zhengs* via clinical observation of septic patients and reports of SARS treatment [11, 13–16]. Two main types of TCM *Zhengs* were finalized: pathogen excess (*邪實證*) and human body deficiency (*正虛證*) [25–27].

The pathogen-excess type manifests in early phases of sepsis, while both infectious agents and host inflammatory reaction are very active. *Qi*-, *Nutrient*-, and *Blood*-phase heat (*氣分熱證*, *營分熱證*, *血分熱證*) were three TCM *Zhengs* finalized in the pathogen-excess type. The *Defense*-phase heat (*衛分熱證*), appearing in very early stage of infection, was not selected since the observed population in our study had already been diagnosed as severe sepsis.

**Figure 1:**
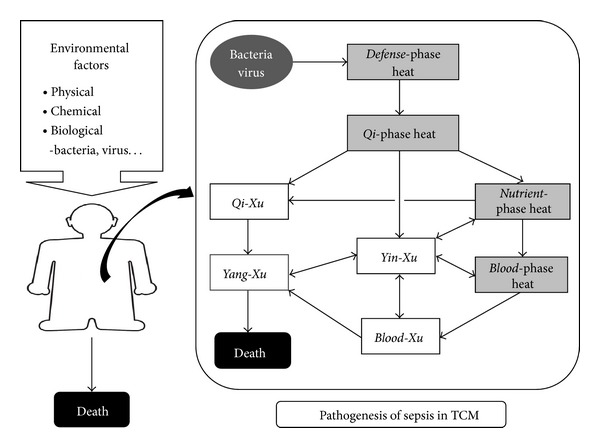
Hypothesis of pathogenesis of sepsis in traditional Chinese medicine. Solid rectangles denote “pathogen-excess type” TCM *Zhengs*. Void rectangles denote “human body-deficiency type” TCM *Zhengs*. Arrows denote possible transition directions and pathways of *TCM Zhengs*.

The human body-deficiency type manifests in late phases of sepsis, in which there is overexpression of inflammatory mediators and multiple impaired functions of body organs [28]. Along with disease progress, pathological heat in *Qi*-, *Nutrient*-, and *Blood*-phase consume *Qi-*,* Blood, Yin, and Yang*, four basic elements to maintain body functions in TCM perspective. Therefore, *Qi-*, *Blood-, Yin-*, and *Yang-Xu* (*氣虛證*, *血虛證*, *陰虛證*, *陽虛證*) were the four TCM *Zhengs* finalized in human body-deficiency type. Finally, experts' opinions on the hypothesis of pathogenesis in TCM reached consensus.  Figure 1 illustrates a hypothesis of possible transition directions and pathways of TCM *Zhengs* from bacterial or viral infection to human death. Diagnostic criteria of each TCM* Zheng* were modified and established from TCM literature, considering easiness to operate in the environment of an Intensive Care Unit (Table 1).

### 2.2. Study Design and Subjects

Prospective observational study was conducted in the medical intensive care units (MICU) of two local community hospitals in Central Taiwan (Nantou and Taichung Hospital, Department of Health, Executive Yuan) from April 2005 to December 2008. Both institutional review boards approved this study. Informed consent was obtained from patients or their family members. Patients who fulfilled diagnostic criteria of severe sepsis or septic shock [29] consecutively admitted to the MICU were enrolled. Those with immunodeficiency, concomitant immunosuppressive therapy, malignancy, pregnancy, severe peripheral vascular disease, or end-stage renal disease were excluded. The study in no way affected patient treatment. All patients had indwelling artery and central line catheters and were mechanically ventilated in pressure controlled modes under continuous analgesic sedation if required. Fluid administration of crystalloids and colloids, dopamine or noradrenaline to maintain mean arterial pressure >65 mmHg, and if needed, dobutamine to maintain cardiac index 4 L/min·m^2^ were given as routine resuscitation therapy for hypotension (systolic blood pressure < 90 mmHg or a reduction of systolic blood pressure by 40 mmHg from baseline). After collection of blood and other suspected infected materials for microbiological analysis, all patients underwent empirical broad-spectrum antibiotic therapy, later adjusted according to culture results.

### 2.3. Sample Size Calculation

As there are few studies in literature on TCM *Zhengs*, sample size was calculated based on our pilot study. During our design stage, we determined that 35 patients in the group with and 53 in the group without *Yang-Xu Zheng* had to provide a power of 90% and 5% of two-sided type 1 error for prevalence of *Yang-Xu Zheng* of 40% in this population to differentiate proportions of patients who died. This was calculated by a two-sided proportion test (*z* test) on the assumption that there was a 40% survival rate in the group with versus 75% survival rate in the group without *Yang-Xu Zheng*.

### 2.4. Data Collection

Whenever a patient diagnosed with severe sepsis or septic shock was admitted to MICU, the physician on duty informed our research team member on call (YYC), who was a specialist and chief of the MICU. Age, gender, TCM *Zheng* diagnosis, and clinical and laboratory measurements for calculation of the APACHE II [17] and cytokine were collected by both reviewing the chart record and examining patients within 24 hours of admission to MICU, and this 24-hour period was considered as “Day 1” in our study. Ambient temperature was controlled at 24°C. To minimize potential observation bias, signs to diagnose TCM *Zhengs* were examined by only one attending physician (YYC) with both Western and Chinese professional training and medical licenses. Patient's survival or death (survivor versus nonsurvivor) in MICU was assessed during a follow-up of a 28-day interval.

### 2.5. Cytokine Measurement

Blood samples were collected from the arterial line for septic patients. Samples were immediately centrifuged in MICU at 1500 rpm for 10 minutes and separated plasma stored at −80°C. Plasma levels of TNF-*α*, IL-6, IL-8, IL-10, and IL-18 were measured by enzyme-linked immunosorbent assay (ELISA) with a commercial kit (R&D systems, Minneapolis, MN) using the manufacture's protocol. All cytokine measurements were performed in the College of Chinese Medicine Laboratory at China Medical University by faculty blinded to clinical data. The samples were assayed with suitable controls for derivation of standard curves.

### 2.6. Statistical Analysis

For continuous variable, data are expressed as mean ± SD and for categorical variables, as numbers with corresponding percentages. Cytokine values were log-transformed to obtain proportionally constant variation and distributed normally. Comparisons between survivor and non-survivor groups were performed via Mann-Whitney *U* test. The Chi-square or Fisher's exact test was used for categorical variables. A multivariate logistic regression was conducted by including all potential variables associated with mortality (i.e., a full model) to assess joint predictive effect on sepsis mortality. Signs of the predictive TCM *Zheng* were analyzed by the full model of logistic regression for selection of independent predictive signs. Statistical calculations were performed using software package SPSS 14.0 (SPSS Inc., Chicago, IL). All comparisons were two-tailed, *P* < 0.05 regarded as statistically significant.

## 3. Results

### 3.1. Characteristics of Study Subjects

A total of 126 sepsis patients were consecutively enrolled: 71 survivors and 55 non-survivors (43.7%) who died within 6.6 ± 5.7 days after MICU admission. Mean age of each group was over 70 years. Male was predominant in the non-survivor group and as risk factor for mortality. Sepsis diagnosis at MICU admission showed no correlation with mortality. APACHE II score was significantly higher among non-survivors. Principal suspected infection source was the respiratory tract, followed by urogenital tract, most infection caused by gram negative bacteria. Six patients with intra-abdominal infection all died within twenty-eight days after admission (Table 2).

### 3.2. Frequency of TCM *Zhengs* between Patients with Severe Sepsis and Septic Shock

Among 126 septic patients, 16 were admitted with diagnosis of severe sepsis, the other 110 with septic shock. Frequency of *Qi-Xu Zheng* was the highest (**68.3%**), followed by *Yang-Xu Zheng* (**51.6%**). Frequency of three pathogen-excess types were between 35.7 and 40.5%. Only 6.3% and 4.8% of patients had *Blood-Xu Zheng* and *Yin-Xu Zheng*, respectively. There was no significance with regard to frequency of all TCM *Zhengs *between patients with severe sepsis or septic shock, although the percentage of *Nutrient*-phase heat and *Qi-Xu Zheng* was much higher in the patients with septic shock (Table 3).

### 3.3. Frequency of TCM *Zhengs* between Survivors and Nonsurvivors

Among three pathogen-excess types, only frequency of *Nutrient*-phase heat *Zheng* was significantly higher in the non-survivor group; in the human body-deficiency types, incidence of both* Qi- *and *Yang-Xu Zheng* was higher in non-survivors (Table 4).

### 3.4. Independent Predictors for Sepsis Mortality

Although frequency of *Qi-Xu Zheng *was also significantly higher in the non-survivor group, it was not included in multivariate analysis model due to its high collinearity with *Yang-Xu Zheng*. While site of infection also has prognostic value [6], intra-abdomen infection was excluded from multivariate analysis model because there was no case in the survivor group. Multivariate logistic regression was performed by including age, gender, APACHE II score, *Nutrient*-phase heat, and *Yang-Xu Zheng* in the model. Only *Yang-Xu Zheng* proved statistically significant in the full model, which had a prediction rate of 70.6% (Table 5). Next, in order to evaluate discrimination capacity of *Yang-Xu Zheng*, we compared APACHE II scores and plasma values of host reactive cytokine of TNF-*α*, IL-6, IL-8, IL-10, and IL-18 between patients with and without *Yang-Xu Zheng*. APACHE II score and values of host reactive cytokines were all significantly higher in the septic patients with *Yang-Xu Zheng* (Table 6). Finally, five diagnostic signs of *Yang-Xu Zheng* were analyzed by multivariate logistic regression. Cool extremities and weak pulse emerged as significant portents for sepsis mortality in the full model, with prediction rate of 74.6% (Table 7).

## 4. Discussion

To our knowledge, this is the first study to evaluate whether TCM *Zheng* diagnosis can be adopted as an early predictor for sepsis mortality. Our prospective observational results indicate (1) *Yang-Xu Zheng* serves as an early predictor for sepsis outcome, since patients with it show higher APACHE II scores; and (2) in cases without it, these host response cytokines are reported as significantly lower: TNF-*α*, IL-6, IL-8, IL-10, and IL-18. Furthermore, cool extremities and weak pulse, two diagnostic signs of *Yang-Xu Zheng,* were also cited as accurate predictors for sepsis mortality and can be adopted for use by Western physicians in their clinical practice.

The most challenging task of our study was to draw the hypothesis of pathogenesis of sepsis from TCM perspective. Experts debated whether to adopt the theory of *Zhang Zhongjing *(*張仲景*) or *Ye Tianshi *(*葉天士*) or to integrate both. The issue was resolved by in-depth literature review, along with discussion of clinical observations and experiences. All experts agreed to integrate these theories into pathogenesis of sepsis, since (1) both theories originate from *Huangdi's Internal Classic* (*黃帝內經*) [25–27] and (2) manifestations of septic patients cover signs and symptoms described in *Treatise on Cold Damage Diseases (Shanghanlun)* and *Treatise on Warm Heat Disease (Wenrelun)*.

Results show complexity of disease progress and TCM *Zheng* transitions in septic patients, who can manifest diverse TCM *Zhengs* simultaneously. The combination can be two *Zhengs* in the pathogen-excess type or human body-deficiency type, or one *Zheng* from pathogen-excess with another from human body-deficiency type; this combination was most frequent. Predominant combination of* Zhengs* reflected both inflammatory reaction caused by the invading pathogen and impaired organ function resulting from cytokine storm and cardiovascular derangements [28].

In constructing the hypothesis of sepsis in TCM, we thought of *Yang-Xu* as the key pathological factor in determining mortality outcome, since *Yang* is energy to maintain body function. This was affirmed by finding *Yang-Xu Zheng* as an independent predictor of mortality. Although the Body Constitution Questionnaire (BCQ+) developed by our team aims at assessing *Yang-Xu* status of the body [30, 31], it is not fitting to measure the *Yang-Xu* status of septic patients in this study. BCQ+ measures the relatively stable physiological condition of the body, but signs and symptoms of sepsis vary quite rapidly under pathological interaction of pathogen virulence and host response [32].

Overwhelming host responses to pathogens may cause inappropriate inflammatory mediator secretion and a variety of acute insults leading to septic shock. Our study, comparing APACHE II score and the host response cytokines between patients with and without *Yang-Xu Zheng, *revealed that patients with *Yang-Xu Zheng* had significantly higher APACHE II scores and host response cytokine values. TCM diagnosis *Yang-Xu Zheng *can thus discriminate septic patients into two groups and help to identify septic patients with higher disease severity and impaired physiological function.

Likewise, cool extremities and weak pulse, both diagnostic signs of *Yang-Xu Zheng, *were found significant prognostic predictors of sepsis mortality. In this study, these diagnostic signs were observed in the postresuscitation phase of septic patients. Even under effects of fluid supplement and inotropic medication, all four distal extremities were still obviously cool to the examiner's hand. This finding concurs with reports from Kaplan et al. [33] and Lima et al. [34]. During examination of the radial artery (side without arterial line insertion), pulse was weak and blood flow easily blocked by fingertip compression. Pulse diagnosis enables TCM physicians to evaluate cardiac output and systemic vascular resistance approximately. In severe sepsis and septic shock, myocardial dysfunction and cardiovascular derangements often manifest [35]. Cool extremities and weak pulse, two signs of peripheral hypoperfusion, are easily assessed and can also be applied by Western physicians to identify septic patients at a higher risk of death within seconds.

In this study, only 6.3% and 4.8% of the patients were found to have *Blood-Xu Zheng* and *Yin-Xu Zheng*, respectively. This may be because (1) our patients were observed in post-resuscitation phase: fluid supplement treatment improved pathological status of *Yin-Xu* and *Blood-Xu*; Yan et al. also noted light tongue color instead of typical crimson tongue in SARS patients after fluid resuscitation [12]; and (2) TCM *Zheng* diagnosis was performed within the first 24 hours after admission: signs and symptoms of *Blood-Xu Zheng* and *Yin-Xu Zheng *did not develop in this period yet.

There are study limitations to note. First, our treatment of septic shock followed the standardized protocol of the hospital, which may not be universal. Since our patients were examined in post-resuscitation phase, effects of sedation and analgesics can not be ruled out. Yet this study provides information on MICU outcomes of patients admitted with severe sepsis and septic shock to local community hospitals in East Asia.

Second, we defined the first 24 hours after admission to MICU as the “Day 1” in our study. It was to be expected that there would be inherent lead-time bias in this study, with septic patients admitted from either the emergency department or inpatient ward. The lead-time bias may underlie observational differences between groups; we suggest that this does not reduce the value of our observations at the point at which decisions about the choice of further hemodynamic support were made.

Third, this study can be criticized for potential observer bias but is supported by other factors, such as both disease severity scores and cytokine values consistent with findings and outcomes.

Fourth, TCM *Zhengs* to diagnose the septic patients were analyzed and singled out from composite *Zhengs* in TCM literatures. Diagnostic criteria were selected after considering difficulties in clinical observation, so only the signs and symptoms that can be observed in the septic patients were included. Since diagnosis *Nutrient*-phase heat, *Qi-Xu*, and *Yang-Xu Zheng* significantly differed between survivors and non-survivors, TCM *Zhengs* and their diagnostic criteria still have the clinical feasibility. With frequency of *Nutrient*-phase heat*, Qi-Xu, *and* Yang-Xu Zheng *higher in non-survivors, further clinical study should evaluate TCM treatment for *Nutrient*-phase heat*, Qi-Xu,* and* Yang-Xu Zheng *in the patients with severe sepsis and septic shock.

## 5. Conclusions

Early identification of patients at high risk of death is a critical issue in management of sepsis. Highly variable signs and symptoms of sepsis emanate from the complex immune reaction and host response. Since TCM *Zheng* diagnosis is made after analyzing all signs and symptoms gathered,* Yang-Xu Zheng* serves as independent and significant prognostic predictor of patients with severe sepsis and septic shock. There were significant differences in APACHE II scores and plasma values of host response cytokine: TNF-*α*, IL-6, IL-8, IL-10 and IL-18 between patients with and without *Yang-Xu Zheng*. These findings provide clinical evidence that TCM* Zheng *diagnosis can be applied to stratify severity and prognosis of septic patients.

## Figures and Tables

**Table 1:** Diagnostic criteria of traditional Chinese medicine (TCM) *Zhengs* in severe sepsis and septic shock.

TCM *Zheng *		Diagnostic criteria				
Type	*Zheng *	Body temperature	Signs^a^		Pulse	Tongue
Pathogen-excess type	*Qi*-phase heat	Fever (BT > 38°C)	Sweating Thirsty Nausea/vomiting Abdominal distension Abdominal pain Constipation Diarrhea			With yellowish coating
	*Nutrient*-phase heat		Delirium		Rapid (HR > 100 beats/min)	Red
	*Blood*-phase heat		Hemorrhage^b^ Petechial or purpuric rash Convulsion			Dark-red crimson

Human body-deficiency type	*Qi-Xu *		Fatigue lethargy	Cool extremities edematous limbs	Weak	—
	*Yang-Xu *					
	*Blood-Xu* *Yin-Xu *	—	Pale		Threadlike	Light-red Red
			Night sweating Thirsty		

^a^Each *Zheng *was diagnosed when either sign appeared.

^b^Bleeding in any part of the body was included, except the skin.

**Table 2:** Demographic and clinical characteristics of septic patients.

Variable	Survivor (*n* = 71)	Nonsurvivor (*n* = 55)	*P* value
Age (yr)	74.7 ± 11.9	73.2 ± 16.6	NS
Gender (male)	38 (53.5%)	39 (70.9%)	<0.05
Survival time (days)		6.6 ± 5.7	
Diagnosis at ICU admission			
Severe sepsis	12 (16.9%)	4 (7.3%)	NS^a^
Septic shock	59 (83.1%)	51 (92.7%)	NS
Severity scoring			
APACHE II	28.5 ± 7.6	31.6 ± 7.7	0.02
Source of infection^b^			
Respiratory tract	38 (53.5%)	26 (47.3%)	NS
Urogenital tract	39 (54.9%)	22 (40.0%)	NS
Liver and biliary tract	2 (2.8%)	4 (7.3%)	NS^a^
Intra-abdomen	0 (0.0%)	6 (10.9%)	<0.01^a^
Cutaneous/soft tissue	5 (7.0%)	1 (1.8%)	NS^a^
Others/unknown	3 (4.2%)	6 (10.9%)	NS^a^
Documented microbial agent^b^			
Gram positive	12 (16.9%)	11 (20.0%)	NS
Gram negative	50 (70.4%)	36 (65.5%)	NS
Fungus	4 (5.6%)	4 (7.3%)	NS^a^

Survivor/nonsurvivor: septic patient alive/dead 28 days after admission to medical intensive care unit.

APACHE II: Acute Physiology and Chronic Health Evaluation II.

Continuous data are presented as Mean ± SD.

Categorical data are presented as number of patients (percentages).

Values have been calculated using Chi-square test and Mann-Whitney *U* test.

^a^Calculated using Fisher's exact test.

^b^Values total more than 100%, since patients could have more than one condition.

*P* < 0.05 statistically significant.

NS: not significant.

**Table 3:** Frequency of TCM *Zhengs* between patients with severe sepsis and septic shock.

TCM *Zheng* ^a^	Total (*n* = 126)	Severe sepsis (*n* = 16)	Septic shock (*n* = 110)	*P* value^b^
Pathogen-excess type				
*Qi*-phase heat	47 (37.3%)	5 (31.3%)	42 (38.2%)	NS^c^
*Nutrient*-phase heat	51 (40.5%)	5 (31.3%)	46 (41.8%)	NS^c^
*Blood*-phase heat	45 (35.7%)	5 (31.3%)	40 (36.4%)	NS^c^
Human body-deficiency type				
*Qi-Xu *	86 (68.3%)	9 (56.3%)	77 (70.0%)	NS
*Yang-Xu *	65 (51.6%)	8 (50.0%)	57 (51.8%)	NS
*Blood-Xu *	8 (6.3%)	1 (6.3%)	7 (6.4%)	NS^c^
*Yin-Xu *	6 (4.8%)	1 (6.3%)	5 (4.5%)	NS^c^

Categorical data are presented as number of patients (percentages).

Values have been calculated using Chi-square test.

^a^Values total more than 100%, since patients could have more than one condition.

^b^Comparison between patient with severe sepsis and septic shock.

^c^Calculated using Fisher's exact test.

*P* < 0.05 statistically significant.

NS: not significant.

**Table 4:** Frequency of each TCM *Zheng* between the survivors and nonsurvivors.

TCM *Zheng* ^a^	Survivor (*n* = 71)	Nonsurvivor (*n* = 55)	*P* value
Pathogen-excess type			
*Qi*-phase heat	27 (38.0%)	20 (36.4%)	NS
*Nutrient*-phase heat	23 (32.4%)	28 (50.9%)	0.04
*Blood*-phase heat	22 (31.0%)	23 (41.8%)	NS
Human body-deficiency type			
*Qi-Xu *	41 (57.7%)	45 (81.8%)	<0.01
*Yang-Xu *	23 (32.4%)	42 (76.4%)	<0.01
*Blood-Xu *	5 (7.0%)	3 (5.5%)	NS^b^
*Yin-Xu *	5 (7.0%)	1 (1.8%)	NS^b^

Categorical data are presented as number of patients (percentages).

Values have been calculated using Mann-Whitney *U* test and Chi-square test.

^a^Values total more than 100%, since patients could have more than one condition.

^b^Calculated using Fisher's exact test.

*P* < 0.05 being statistically significant.

NS: not significant.

**Table 5:** Multivariate analysis of independent predictor for sepsis mortality.

Variable	Prediction rate: 70.6%				
	*B*	Wald	Relative risk	95% CI	*P* value
Age	−0.01	0.77	0.99	0.96–1.02	NS
Gender	0.33	0.55	1.39	0.59–3.29	NS
APACHE II	0.03	1.11	1.03	0.98–1.01	NS
*Nutrient*-phase *Zheng *	0.31	0.51	1.36	0.58–3.16	NS
*Yang-Xu Zheng *	1.75	16.62	5.74	2.48–13.3	<0.01

Multivariate logistic regression analysis was performed by “enter” method.

APACHE II: Acute Physiology and Chronic Health Evaluation II.

CI: confidence interval.

*P* value < 0.05 being statistically significant.

**Table 6:** APACHE II score and host reactive cytokine levels between septic patients with and without *Yang-Xu Zheng*.

	With *Yang-Xu Zheng* (*n* = 65)	Without *Yang-Xu Zheng* (*n* = 61)	*P* value
Severity scoring			
APACHE II	31.8 ± 7.8	27.8 ± 7.1	<0.01
Host reactive cytokine			
TNF-*α* ^a^	1.6 ± 0.5	1.4 ± 0.5	<0.01
IL-6^a^	3.3 ± 0.9	2.8 ± 0.8	<0.01
IL-8^a^	2.5 ± 0.6	2.2 ± 0.5	<0.01
IL-10^a^	2.3 ± 0.7	2.0 ± 0.7	<0.01
IL-18^a^	2.9 ± 0.2	2.8 ± 0.3	<0.01

APACHE II: Acute Physiology and Chronic Health Evaluation II.

Continuous data are presented as Mean ± SD.

Values have been calculated using Mann-Whitney *U* test.

TNF: tumor necrosis factor.

IL: interleukin.

^a^Based on log transformed values (pg/mL).

*P* < 0.05 statistically significant.

NS: not significant.

**Table 7:** Diagnostic signs of *Yang-Xu Zheng* as independent predictors of sepsis mortality.

Variable	Prediction rate: 74.6%				
	*B*	Wald	Relative risk	95% CI	*P* value
Fatigue	0.34	0.14	1.41	0.24–8.32	NS
Lethargy	−1.35	2.96	0.26	0.06–1.21	NS
Cool extremities	1.94	17.94	6.97	2.84−17.12	<0.01
Edematous limbs	0.51	1.36	1.66	0.71–3.92	NS
Weak pulse	1.35	5.64	3.86	1.27–11.77	0.02

Multivariate logistic regression analysis was performed by “enter” method.

CI: confidence interval.

*P* < 0.05 being statistically significant.
